# Comparative analysis of function and interaction of transcription factors in nematodes: Extensive conservation of orthology coupled to rapid sequence evolution

**DOI:** 10.1186/1471-2164-9-399

**Published:** 2008-08-27

**Authors:** Wilfried Haerty, Carlo Artieri, Navid Khezri, Rama S Singh, Bhagwati P Gupta

**Affiliations:** 1 Department of Biology, McMaster University, Hamilton, ON L8S 4K1, Canada

## Abstract

**Background:**

Much of the morphological diversity in eukaryotes results from differential regulation of gene expression in which transcription factors (TFs) play a central role. The nematode *Caenorhabditis elegans *is an established model organism for the study of the roles of TFs in controlling the spatiotemporal pattern of gene expression. Using the fully sequenced genomes of three *Caenorhabditid *nematode species as well as genome information from additional more distantly related organisms (fruit fly, mouse, and human) we sought to identify orthologous TFs and characterized their patterns of evolution.

**Results:**

We identified 988 TF genes in *C. elegans*, and inferred corresponding sets in *C. briggsae *and *C. remanei*, containing 995 and 1093 TF genes, respectively. Analysis of the three gene sets revealed 652 3-way reciprocal 'best hit' orthologs (nematode TF set), approximately half of which are zinc finger (ZF-C2H2 and ZF-C4/NHR types) and HOX family members. Examination of the TF genes in *C. elegans *and *C. briggsae *identified the presence of significant tandem clustering on chromosome V, the majority of which belong to ZF-C4/NHR family. We also found evidence for lineage-specific duplications and rapid evolution of many of the TF genes in the two species. A search of the TFs conserved among nematodes in *Drosophila melanogaster*, *Mus musculus *and *Homo sapiens *revealed 150 reciprocal orthologs, many of which are associated with important biological processes and human diseases. Finally, a comparison of the sequence, gene interactions and function indicates that nematode TFs conserved across phyla exhibit significantly more interactions and are enriched in genes with annotated mutant phenotypes compared to those that lack orthologs in other species.

**Conclusion:**

Our study represents the first comprehensive genome-wide analysis of TFs across three nematode species and other organisms. The findings indicate substantial conservation of transcription factors even across distant evolutionary lineages and form the basis for future experiments to examine TF gene function in nematodes and other divergent phyla.

## Background

The growing availability of the whole-genome sequences of eukaryotes has accelerated large-scale functional studies to understand the mechanisms of animal development and evolution [1-4]. Many of these studies have highlighted the importance of regulatory evolution and the fundamental role that transcription factors (TFs) play in this process. Alterations in TF function and regulation are linked to phenotypic variation [5-7] as well as numerous pathologies, including cancers [8,9]. Therefore, a detailed analysis of sequence and function of TFs across animal phyla will provide important information about their evolutionary patterns, thereby increasing our ability to understand the molecular basis of diseases and organismal complexity. The nematode *Caenorhabditis elegans *serves as a powerful model organism to unravel TF function due to the wealth of available resources and the ease with which it can be reared, maintained, and manipulated in the laboratory [10]. The completion of its genome sequence has aided in the design of large-scale experiments that are beginning to elucidate the complexity of transcriptional regulation and gene interaction networks in multicelllular eukaryotes [11,12]. The recent releases of the genome sequence of two other *Caenorhabditid *species, *C. briggsae *[13] and *C. remanei *[14], provide an excellent opportunity for genome-wide study of the conservation and evolution of transcription factors across nematodes. These three species are estimated to have shared a common ancestor between 20–120 million years ago [13-15] and while they are morphologically similar, studies have shown differences in development and behavior [16].

As a first step in facilitating the comparative study of TFs in nematodes, we have compiled an updated list of putative TF genes in *C. elegans *and used it to identify orthologs in *C. briggsae *and *C. remanei*. Our results show that two-thirds of all *C. elegans *TF genes have 3-way one-to-one best reciprocal orthologs in the other two species, whereas the remaining third are either species-specific paralogs or too divergent to assign proper orthologous relationships. We observed that among *Caenorhabditid *species, although TF genes have a greater sequence divergence than the non-TF genes, they exhibit significantly more detectable interspecific orthologs than non-TF genes. We also identified 150 best reciprocal orthologs of the TF genes conserved among nematodes in fruit fly (*Drosophila melanogaster*), mouse (*Mus musculus*), and human (*Homo sapiens*) many of which are associated with known disorders. We also examined the relationship between gene function and interactions, the results of which demonstrate that conserved TF genes exhibit a significantly greater number of interactions and are more likely to be associated with mutant phenotypes when compared to those that lack detectable orthologs. Our findings provide a framework for future studies of nematode TFs and facilitate the development of resources allowing us to study morphological and developmental diversity in metazoans.

## Results

### The *C. elegans *TF gene set

As a first step in the identification of TFs in *Caenorhabditid *species, we generated an updated list of putative *C. elegans *TF genes by searching its annotated genome sequence (Wormbase WS173 release) [17] for gene ontology (GO) terms associated with transcription factors. This led to the identification of 1271 putative TF genes (Table 1). Since our criteria for selecting a TF was the presence of a well-defined DNA binding domain that selectively modulates gene transcription (for example, bHLH or homeobox), we manually inspected the above list of putative TFs. This allowed us to reject 564 genes as false positives since these encode factors that are associated with the basal transcriptional apparatus (for example, DNA polymerases), chromatin alterations, DNA packaging (histones), as well as entries that were incorrectly curated in Wormbase (Additional files 1, 2, 3). To the remaining genes (707), we added 281 TF encoding genes found in published literature and other public database entries that were not identified in our initial search (See Materials and Methods). The final *C. elegans *TF set included a total of 988 genes (Table 2 and additional file 4), of which 917 are shared with the previously annotated *C. elegans *TF set (wTF2.0, 934 genes) [18]. The 17 genes in the wTF2.0 set that are not shared in our updated set either lack a known DNA binding domain or are annotated as pseudogenes (Additional file 5). The increased number of genes in the present TF set likely results from the availability of annotations published since the compilation of wTF2.0.

**Table 1:** GO term-based searches of TF genes in *C. elegans*.

GO ID	Term	TF genes	Unique
0003700	Transcription Factor activity	614	6
0043565	DNA binding, sequence specific	515	6
0003677	DNA binding	858	352
0030528	Transcription regulator activity	75	9
0006355	Regulation of transcription, DNA dependent	768	78
0045449	Regulation of transcription	199	19
0000122	Negative regulation of transcription from RNA pol II promoter	8	4
004544	Positive regulation of transcription from RNA pol II promoter	24	10

The table lists numbers of all TF genes as well as those uniquely identified by each of the terms.

**Table 2:** The breakdowns of TF genes in each of the nematode species genomes based on various search categories.

**Search method**	**Number of TF genes**		
	
	***C. elegans***	***C. briggsae***	***C. remanei***
GO term-based	707	ND	NA
Orthologs (InParanoid and reciprocal BLAST)	ND	713	703
Manual curation	281	NA	NA
HMM alignments	ND	282	390

**TOTAL:**	**988**	**995**	**1093**

NA: not applicable, ND: not done.

### Identification of transcription factors in nematodes and other phyla

**Figure 1:**
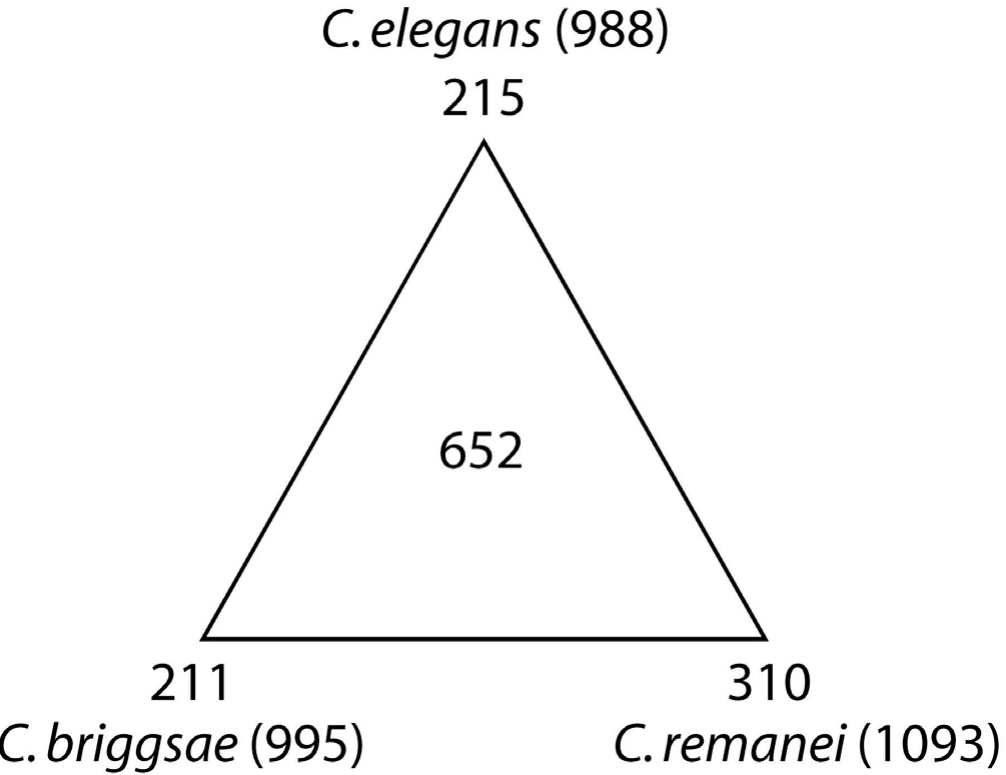
**TF-encoding genes in *C. elegans*, *C. briggsae *and *C. remanei*.** The total number of TF genes in each of the species is given inside the brackets. The numbers of divergent TF genes and those conserved among the three nematode species are shown along the vertices and inside of the triangle, respectively.

We used the newly defined *C. elegans *TF set to search for homologs in the fully sequenced genomes of *C. briggsae *(CB3 release) and *C. remanei *(11/29/2005 release) [17,19,20]. We used InParanoid [21] to identify 713 and 703 best reciprocal hit orthologs in *C. briggsae *and *C. remanei*, respectively (Table 2, see Material and Methods). To these lists, we added 282 *C. briggsae *and 390 *C. remanei *putative TF genes that were identified through Hidden Markov Model (HMM)-based searches [22]. Altogether, a total of 995 and 1093 potential TF encoding genes were identified in *C. briggsae *and *C. remanei*, respectively (Table 2 and additional files 6 and 7). Among the TF orthologs in the three nematode species, we identified 652 genes that exhibit a 3-way best reciprocal BLAST orthologous relationship (hereafter referred to as the nematode TF set) (Figure 1). The proportion of *C. elegans *TF genes with detectable orthologs in *C. briggsae *(713/995, 71.7%) is significantly higher compared to the proportion of all conserved genes between the two species (12858/20621, 62.4%) [13] (χ^2 ^= 7.56, df = 1, *p *= 6.0 × 10^-3^), which may indicate strong selective pressure to maintain these genes.

To examine the evolutionary conservation of the nematode TF set of genes in other phyla, we searched for their orthologs in the genomes of fruit fly(*D. melanogaster*), mouse (*M. musculus*), and human (*H. sapiens*). Using the InParanoid database [23] we identified a total of 150 TFs that exhibit reciprocal orthologous relationships between three nematode species and are conserved in fly, mouse, and human (Additional files 4 and 8).

### Coding sequence divergence in nematode TF genes

Best-hit reciprocal orthologs could not be identified for 215 TF genes in *C. elegans*, 211 in *C. briggsae*, and 310 in *C. remanei *(Figure 1 and additional files 4, 6, and 7). It should be pointed out that *C. briggsae *and *C. remanei *TF genes are based on computational predictions and that the *C. remanei *genome has yet to be assembled; hence while many of the TF genes without detectable orthologs may have arisen by lineage-specific gene duplication, others could result from incomplete annotation of the *C. briggsae *and *C. remanei *genomes. Therefore, the actual number of divergent TF genes in these species is likely to be smaller than the numbers we have estimated. To further study this set of genes in *C. briggsae *(211), we searched for their closest homologs in *C. elegans*. This revealed 30 genes with weak sequence similarity (BLASTP E-value > 10^-10^) suggesting that these most likely represent candidate *C. briggsae*-specific TF genes (Additional file 9). The remaining 181 appear to be species-specific paralogs, of which 69 are zinc finger-C4/nuclear hormone receptor (ZF-C4/NHR) family members (see below).

**Figure 2:**
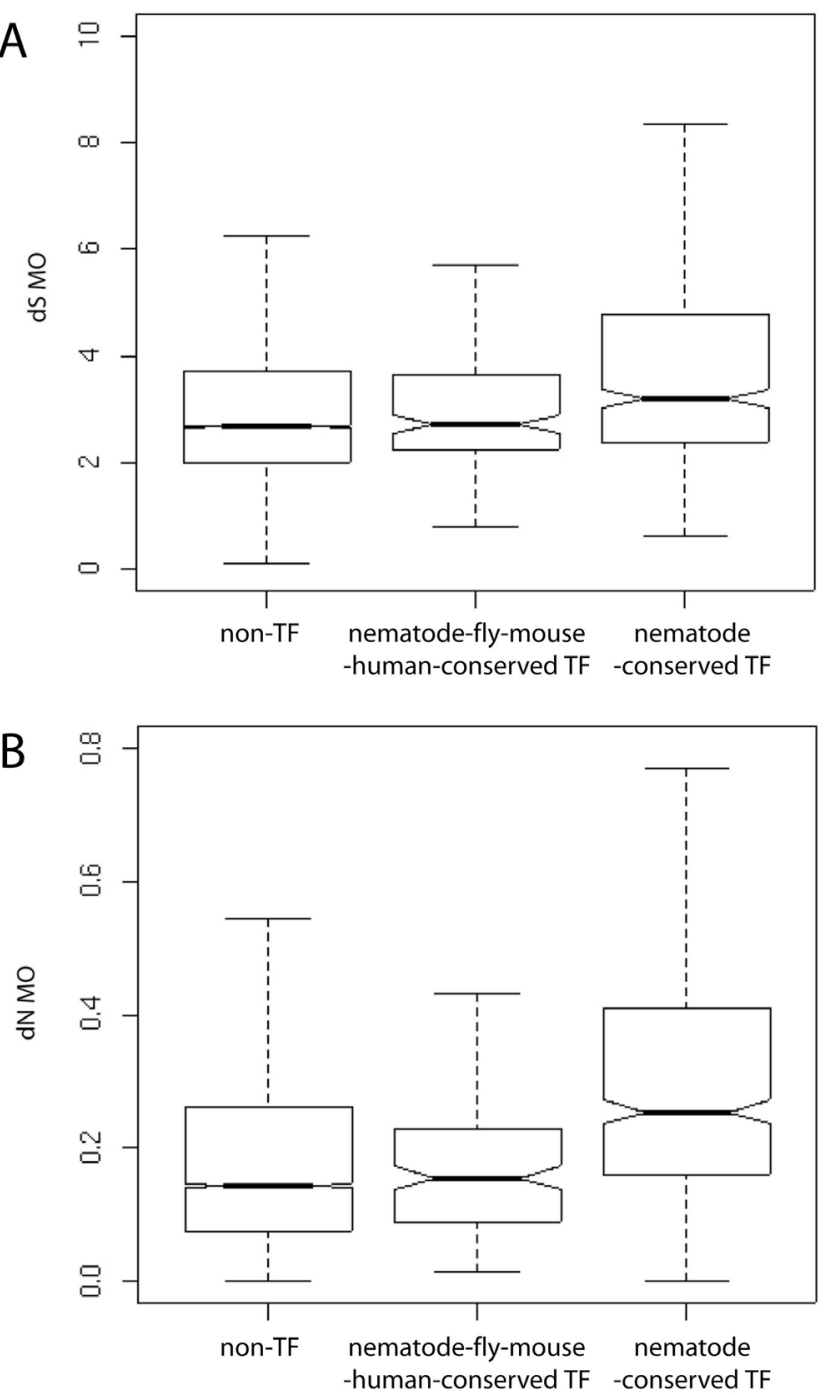
**Sequence divergence of transcription factors in *C. elegans*.** Rates of synonymous substitutions per synonymous site (*d*_S_) (A) and non-synonymous substitutions per non-synonymous site (*d*_N_) (B) as calculated under model 0 in PAML (Yang) for non-TF (10,827), TF genes conserved in nematodes, fly, mouse, and human (150) and TF genes conserved among the three nematode species (652) are shown. The boxplot indicates the first and third quartiles and the dotted lines the 5th and 95th percentiles. The notches indicate the level of uncertainty associated with the median.

Previous studies in humans and other organisms have shown that TF genes tend to evolve more rapidly than non-transcription factor (non-TF) genes [24-26], therefore we performed a similar analysis in nematodes by analyzing their coding sequence divergence and comparing it to non-TF genes. Due to the large divergence times between the three species [13,15], the rate of synonymous substitution per synonymous site (*d*_S_) for many genes is likely to be saturated (*d*_S _> 3, Figure 2A). Therefore we restricted our analysis to the rate of non-synonymous substitution per non-synonymous site (*d*_N_), which does not show such saturation [27]. We found a significantly higher *d*_N _for TF genes conserved among nematodes (652) when compared to 3-way conserved non-TF gene orthologs (10,827 genes; Kruskal-Wallis rank sum test, *p *< 2.2 × 10^-16^; Figure 2B), whereas no difference was detected between TF genes with orthologs in nematodes, fly, mouse, and human (150) and non-TF genes (Kruskal-Wallis test, *p *= 0.3498).

### Distribution of TF families in *C. elegans*

**Figure 3:**
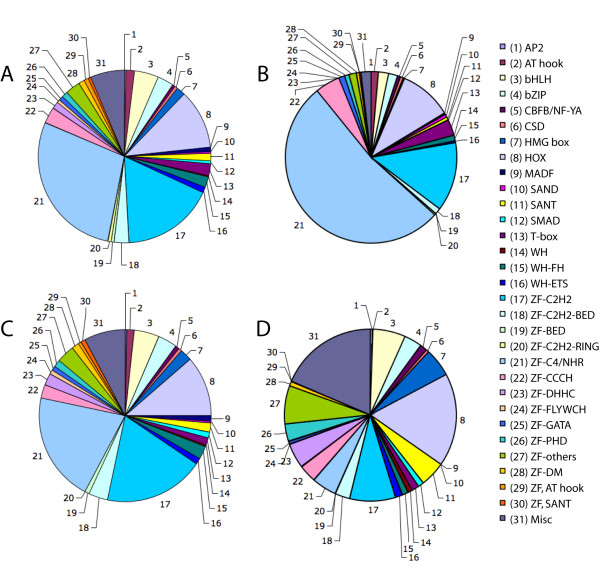
**Distribution of TF gene families in *C. elegans*.** The pie charts show distributions of all (A), divergent (B), nematode-conserved (C), and nematode-fly-mouse-human-conserved (D) TF genes in *C. elegans*. For details on various gene families please refer to Materials and Methods.

We studied the distribution of protein families among *C. elegans *TFs based on known DNA binding domains. This analysis revealed more than 50 distinct families of which 30 were found to contain 5 or more members (Figure 3). No significant difference was observed in the representation of various families between the *C. elegans *set and the set conserved among three nematodes (χ^2 ^= 35.05, df = 30, *p *= 0.2408), indicating that the distributions of TF families in these species may be similar. The majority of genes in *C. elegans *and nematode TF sets (28.6% and 20.5%, respectively) were found to encode the nuclear hormone receptors (NHRs), a C4-type sub-family of zinc finger proteins that play key roles in development and homeostasis [28]. The NHR genes was previously shown to have undergone extensive lineage-specific expansion in *C. elegans *[29]. Besides NHR, HOX genes that regulate cell fate specification and embryogenesis [30] are also among the largest TF families in nematodes (11% of *C. elegans *TF genes, and 11.6% of nematode TF genes) (Figure 3A, C). In contrast, the distribution of TF families among the divergent *C. elegans *gene set (215 genes) differs significantly from that observed among the entire *C. elegans *TF set (χ^2 ^= 83.91, df = 30, *p *= 5.33 × 10^-7^) due to its high proportion of NHR genes (52.6%, Figure 3B). Likewise, the representation of different families among TF genes with orthologs in nematodes, fly, mouse, and human also differs from that of the *C. elegans *TF set (χ^2 ^= 152.27, df = 30, *p *= 0, Figure 3D). Interestingly, the single largest conserved family represented among the orthologs in three different phyla is HOX (17.3%), supporting multiple previous studies indicating the importance of this family among all metazoans [18,30,31].

### Chromosomal distribution of TF genes in *C. elegans *and *C. briggsae*

**Figure 4:**
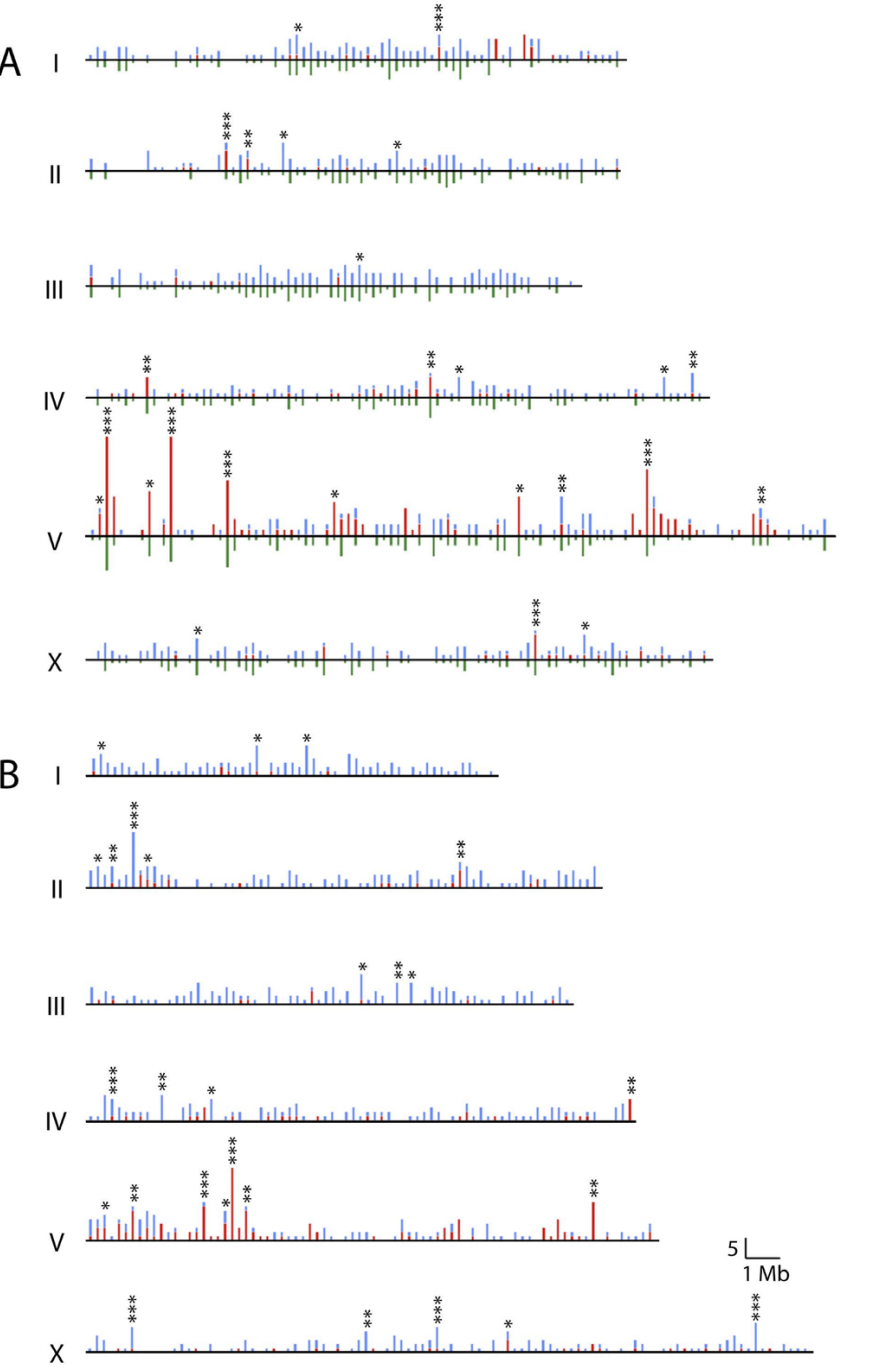
**Chromosomal distribution of TF genes in *C. elegans *(A) and *C. briggsae *(B).** The maps have been plotted by taking all TF genes in non-overlapping 200 kb windows. The color codes are as follows. Red: NHR genes, blue: non-NHR TF genes, green: TF genes conserved among the three nematode species. Gene clustering was analyzed by comparing the numbers of TF and non-TF genes located in each window using a χ^2 ^test. Gene clusters that are significantly enriched have been marked with stars (*: P < 0.05; ** P < 0.01; ***: P < 0.0001).

Studies in *C. elegans *as well as other organisms have shown that genes that are co-expressed and/or functionally related are frequently clustered together on chromosomes [32-36]. To investigate whether TF genes in nematodes exhibit a similar pattern, we plotted the physical locations of *C. elegans *and *C. briggsae *TF genes using non-overlapping windows of 200 kb (the genome of *C. remanei *has not yet been assembled and therefore was not used in this analysis). Figure 4 shows that TF genes in *C. elegans *and *C. briggsae *as well as those that are conserved among nematodes are non-randomly distributed on chromosomes. A total of 183 *C. elegans *TF genes were found to be located in 25 distinct clusters (marked with stars in Figure 4A, Table 3). A similar pattern was observed in *C. briggsae *(184 genes in 27 clusters) (Figure 4B and Table 3). Chromosome V carries highest number of clusters (and genes) in both species (*C. elegans*: 97 genes in 10 clusters; *C. briggsae*: 64 genes in 7 clusters) (Table 3) that are primarily composed of NHR family members (92% in *C. elegans *and 84% in *C. briggsae*, red bars in Figure 4).

**Table 3:** Chromosome-wise breakdown of TF gene clusters in *C. elegans *and *C. briggsae*.

**Chromosome**	***C. elegans***		***C. briggsae***	
		
	**Number of clusters**	**Number of genes**	**Number of clusters**	**Number of genes**
1	2	12	3	19
2	4	24	5	34
3	1	5	3	17
4	5	29	4	21
5	10	97	7	64
X	3	16	5	29

**TOTAL**	**25**	**183**	**27**	**184**

**Figure 5:**
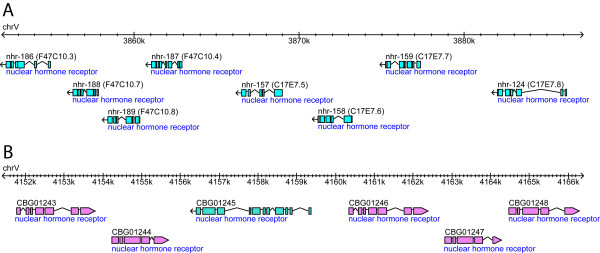
**Tandem arrays of NHR genes in *C. elegans *and *C. briggsae*.** The snapshots of the genomic regions, visualized by Wormbase genome browser, show 8 genes in *C. elegans *and 6 in *C. briggsae*. The colors of the open reading frames indicate their orientation (blue: leftward, pink: rightward).

The analysis of the chromosomal distribution of TF genes also revealed that many members of the large TF families, such as ZF-C4/NHR, ZF-C2H2, T-box and HOX are arranged in perfect tandem arrays (defined as having a contiguous repetition of TF genes) (267 *C. elegans *genes in 103 arrays, 235 *C. briggsae *genes in 107 arrays), the largest of which consists of 8 NHR genes in *C. elegans *and 6 NHR genes in *C. briggsae *(Figure 5 and additional files 10 and 11). Although the majority of such arrays consists of genes of the same TF family (76.7% in *C. elegans *and 67.3% in *C. briggsae*), less than half of all genes found in such arrays have best-reciprocal hit orthologs between the two species (31.6% in *C. elegans*, 47.2% in *C. briggsae*) (Additional files 10 and 11) suggesting significant lineage-specific duplication and expansion of the tandem arrays.

### Evolution of the Nuclear Hormone Receptor family in nematodes

Our findings extend Robinson-Rechavi et al.'s analysis of the extensive lineage-specific expansion of NHR genes in *C. elegans *[29] to the other two *Caenorhabditid *species (283, 232, and 256 NHR genes in *C. elegans*, *C. briggsae*, and *C. remanei*, respectively). The sequence analyses revealed a total of 134 NHR genes having 3-way best-reciprocal orthologs among the nematode species (Additional files 4, 6, and 7). The remaining NHRs are composed of what appear to be lineage-specific paralogs and those that have diverged sufficiently in sequence such that orthologous relationships could no longer be assigned.

We constructed a phylogenetic tree of the nematode NHR family members (437 genes, see Materials and Methods) to study their inter– as well as intra-specific relationships. The most striking feature of the phylogeny is the frequent presence of several closely related NHRs located tandemly on the same chromosome (Additional file 12). Such groupings suggest the presence of extensive tandem duplications, which could explain the mechanism behind the expansion of the NHR gene family, and perhaps the independent occurrence of some NHR genes in the lineages of each of these species. In the case of *C. elegans *NHRs, we found at least 15 distinct groups on chromosome V including 7 that are located in one large cluster of the phylogeny (Additional file 12).

The presence of NHRs in chromosomal clusters prompted us to study their distribution in further detail. We identified a total of 47 tandem arrays composed of contiguous repetitions of NHR genes in *C. elegans*, which are found on all chromosomes with the exception of chromosome III (Additional file 10). These include 10 arrays that are comprised of 5 or more genes, all of which are located on chromosome V. A similar analysis in *C. briggsae *identified 30 NHR arrays having 6 or fewer genes (Additional file 11). In total, 9 NHR arrays were partially or completely conserved between *C. elegans *and *C. briggsae*. One of these arrays, for instance, consists of 7 genes in *C. elegans *(*nhr-136, nhr-153, nhr-154, nhr-206, nhr-207, nhr-208*, and *nhr-209*) and the corresponding 4 in *C. briggsae *(*CBG23383/Cbr-nhr-136, CBG23380/Cbr-nhr-153, CBG23380/Cbr-nhr-154 *and *CBG23379/Cbr-nhr-209*). This suggests that either the array has expanded in *C. elegans *or perhaps lost 3 of the genes in *C. briggsae*. Examination of the *C. remanei *TFs revealed the presence of best reciprocal hit orthologs for all array members found in *C. elegans *with the exception of *nhr-206 *leading us to propose that *nhr-207 *and *nhr-208 *were most likely lost in the *C. briggsae *lineage. This analysis, however, carries a caveat in that the annotations of the *C. briggsae *and *C. remanei *genomes are based on computational predictions and lack experimental validation.

Finally, we found that 7 tandem arrays in *C. briggsae *are composed of NHR genes that lack best reciprocal hit orthologs in *C. elegans *and *C. remanei *(Additional file 11). The largest of these is comprised of 6 NHR genes (*CBG01243, CBG01244, CBG01245, CBG01246, CBG01247, CBG01248*) (Figure 5). These *C. briggsae*-specific arrays may be caused by lineage-specific expansion although the possibility of a selective loss of their orthologs in other species cannot be ruled out.

### Comparison of TF gene sequence conservation and function in *C. elegans*

We investigated the relationship between sequence conservation and function of TF genes in *C. elegans*. From a comprehensive list of 13,647 RNAi phenotypes associated with 4,351 genes [14], we identified 281 TFs that exhibit one or more mutant phenotypes (Additional file 13). These consist of more than half of all TF genes conserved among nematodes, fly, mouse, and human (52.7%, 79 of 150), over one-third of genes conserved among the three nematode species (36.5%, 238 of 652), and one-fifth of the TF genes in *C. elegans *that did not have identifiable orthologs in the other nematode species (20%, 43 of 215). We also determined the number of distinct mutant phenotypes associated with TF genes in each of the above three groups as well as with non-TF genes. This analysis revealed that TF genes conserved among nematodes, fly, mouse, and human are linked to a significantly greater number of mutant phenotypes in *C. elegans *when compared to the other sets (4.38 ± 2.31, 3.36 ± 2.09, 2.91 ± 1.82 and 3.19 ± 1.84 phenotypes per gene for TF genes conserved across phyla, conserved in nematodes, *C. elegans *TF genes without detectable orthologs in the other nematode species and non-TF genes, respectively; Kruskal-Wallis rank sum test, *p *= 8.58 × 10^-3^, 1.8 × 10^-3^, 1.32 × 10^-15^, respectively, after Bonferroni correction). No difference was found in pairwise comparisons between the other gene sets (Kruskal-Wallis rank sum test *p *= 1, in all comparisons after Bonferroni correction).

**Figure 6:**
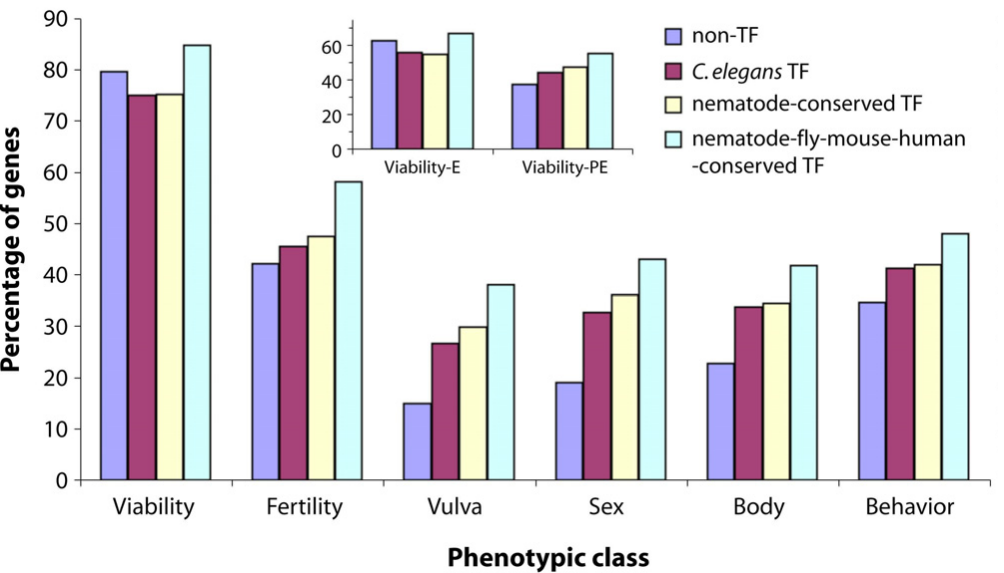
**Functional classification of *C. elegans *TF genes.** The six broad categories are based on the mutant phenotypes in RNAi studies. Non-TF genes have been plotted for comparison. Viability-E and viability-PE are based on the embryonic and post-embryonic stage lethality phenotypes in RNAi assays, respectively. Refer to text for the description of other categories.

To further analyze the roles of *C. elegans *TF genes in specific tissues and developmental processes, RNAi phenotypes were sorted into six broad categories: viability (embryonic and post-embryonic growth and survival), fertility (germline and germ cells), sex (sex determination and reproductive system), vulva (vulval cell proliferation and morphogenesis), body (cuticle, size, and morphology), and behavior (movement and feeding) (Additional files 13 and 14). Among the six categories, "viability" ranks highest in terms of the proportion of TF and non-TF genes (Figure 6). However, it is important to keep in mind that this may be linked to a greater interest in identifying transcription factors that are involved in growth and survival of *C. elegans*. A further sub-classification of this category into "embryonic viability" and "post-embryonic viability" (based on the phenotype when lethality occurs in RNAi-treated animals) revealed that among the "embryonic viability" class TF genes conserved among nematode species are significantly under-represented when compared to the non-TFs (χ^2 ^= 7.39, df = 1, *p *= 6.56 × 10^-3^) (Figure 6), while no difference was observed among the datasets for genes affecting post-embryonic viability (χ^2 ^= 0.77, df = 1, *p *= 0.38). By contrast, the TF genes conserved among nematodes, fly, mouse, and human showed no enrichment for any of these two categories (χ^2 ^= 3.59 and 0.39, df = 1, *p *= 0.059 and 0.53, respectively). Among other categories, we observed an over-representation of mutant phenotypes for nematode-conserved as well as nematode-fly-mouse-human-conserved TF gene sets associated with vulval development (χ^2 ^= 8.24 and 11.75, df = 1, *p *= 4.1 × 10^-3 ^and 6.08 × 10^-4^, respectively) and sex determination and reproductive system-related processes (χ^2 ^= 9.51 and 8.78, df = 1, *p *= 2.04 × 10^-3 ^and 3.59 × 10^-3^, respectively) when compared to the non-TF gene set.

### Phenotypes associated with nematode TF orthologs in fly, mouse, and human

The above findings that more than half of *C. elegans *TF genes conserved across phyla are associated with RNAi phenotypes prompted us to examine their mutant phenotypes in other organisms. We found that 69 (46%) of TF genes conserved among nematodes, fly, mouse, and human are associated with lethal phenotype in fly (Additional file 15). In the case of mouse, out of a total of 81 orthologs for which knock out and mutant phenotypes are described (see Materials and Methods), 75 (92.6%) exhibit defects ranging from mild to gross abnormalities, including lethality (Additional file 15). A similar analysis in human revealed 35 TF genes linked to various diseases and genetic disorders (Table 4). In total, 44 (29.3%) TF genes regulating *C. elegans *development and behavior are also essential either in mouse or human or both. These include 30 genes that control viability in the fruit fly. Overall, 121 (80.7%) TF genes conserved among nematodes, fly, mouse, and human play important roles in at least one of these organisms. This is likely an underestimate due to technical limitations of RNAi experiments (e.g., strains, redundancy of factors or pathways) and that comparisons between organisms involve different experimental approaches (e.g., RNAi in *C elegans *and chromosomal mutations in *D. melanogaster*). Thus, functional studies of conserved TFs in *C. elegans *promise to elucidate mechanisms involved in biological processes conserved across phyla.

**Table 4:** Genetic disorders linked to human TF genes conserved among nematodes, fly, mouse, and human.

***C. elegans *gene**	**Human ortholog**	**Human disorder**
*vab-3*	*Pax6*	Aniridia type II, Peters anomaly with cataract, foveal hypoplasia
*Y38H8A.5*	*FEZF1*	Beckwith-Wiedemann syndrome
*ceh-33*	*SIX1*	Branchiootic syndrome 3
*mab-9*	*TBX20*	Cardiomyopathy, atrial septal defect 1
*tag-192*	*CHD7*	CHARGE syndrome
*ceh-24*	*TITF1*	Congenital hypothyroidism, neonatal respiratory insufficiency
*dve-1*	*SATB2*	Cleft palate isolated
*ceh-14*	*LHX3*	Combined pituitary hormone deficiency 3
*unc-86*	*Pou4f3*	DFNA15 syndrome
*elt-1*	*GATA1*	Dyserythropoietic anemia with thrombocytopenia
*K02H8.1*	*MBNL2*	Dystrophia myotonica 1
*fax-1*	*Nr2e3*	Enhanced s-cone syndrome
*ceh-17*	*PHOX2A*	Congenital fibrosis of the extraocular muscles 2
*ceh-32*	*SIX3*	Holoprosencephaly 2
*sbp-1*	*Srebf1*	Hypercholesterolemia, familial
*lin-28*	*LIN28B*	Hypomyelination and cataract
*alr-1*	*ARX*	Lissencephaly, X-linked, with ambiguous genitalia
*hmg-5*	*Tfam*	Kearns-Sayre syndrome
*cnd-1*	*NEUROD1*	Maturity-onset diabetes of the young
*lim-6*	*LMX1B*	Nail patella syndrome NPS1
*grh-1*	*GRHL2*	Neurosensory deafness 28
*sma-4*	*Smad4*	Pancreatic cancer, Hemorrhagic Telangiectasia Syndrome (HTT)
*nhr-6*	*NR4A2*	PARK14
*ceh-6*	*POU3F3*	Perilymphatic gusher-deafness syndrome
*zag-1*	*ZEB1*	Posterior polymorphous corneal dystrophy 3
*eor-1*	*MYNN*	Promyelocytic leukemia
*R07E5.3*	*Smarcb1*	Rhabdoid tumor
*cbp-1*	*CREBBP*	Rubinstein-taybi syndrome, acute myeloid leukemia
*ceh-43*	*DLX5*	Split-hand/foot malformation
*ing-3*	*ING3*	Squamous cell carcinoma
*ast-1*	*FLI1*	Thrombocytopenia, Paris-Trousseau type
*nhr-64*	*HNF4A*	Maturity-onset diabetes of the young
*tbx-2*	*Tbx2*	Ulnar mammary syndrome
*K02D7.2*	*SNAI2*	Waardenburg syndrome, piebaldism
*F53F8.1*	*KLF3*	Wilms tumor

### Analysis of TF interaction networks in *C. elegans*

**Figure 7:**
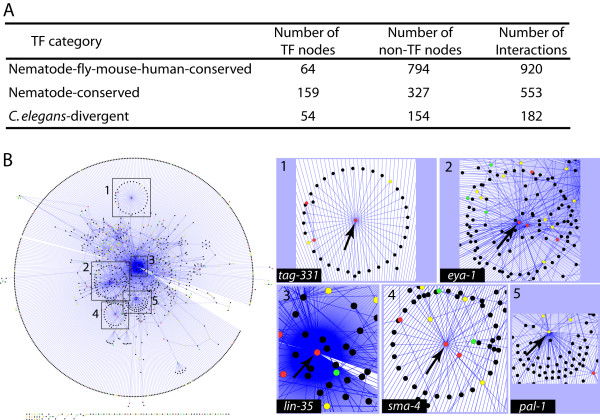
**The interaction network of *C. elegans *TF genes.** (A) The breakdowns of TF nodes, non-TF nodes and gene interactions in the network for each of the TF categories. The *C. elegans*-divergent category refers to TF genes that lack unique reciprocal orthologs in other nematode species. (B) The network exhibits several high degree nodes, five of which – *tag-331, eya-1, lin-35, sma-4*, and *pal-1 *– are boxed and shown at high magnification on the right (marked by arrows). The node colors mark different TF genes (red: conserved among nematodes, fly, mouse, and human; yellow: conserved among the three nematode species; green: *C. elegans*-divergent). The network was visualized by using Cytoscape [81].

To further explore the mechanism of transcription factor function in metazoans, we generated an interaction map of *C. elegans *TF genes based on known physical and genetic interactions [37,38]. The map consists of 1594 interactions involving 277 TF genes and their direct non-TF interactors (Figure 7A and additional file 16). The network appears to be scale free as seen by the presence of several nodes with high degree of connectivity (such as *lin-35*, which shows the highest number of interactions and is connected to more than one-third of all existing nodes; 521 of 1340) (Figure 7B). *lin-35 *is an ortholog of the human *Retinoblastoma *(*Rb*) gene which plays an important role in cell proliferation [39,40]. Among the *lin-35 *interacting genes, 43 (8%) encode TFs, of which 18 have best reciprocal hit orthologs in mouse and human. Other prominent hubs include *pal-1 *(conserved among nematode species), as well as other TF genes with orthologs in nematodes, fly, mouse, and human: *tag-331, eya-1*, and *sma-4 *(Figure 7B). Each of these genes plays important role in *C. elegans *development, and RNAi-mediated knock-downs cause defects such as slow growth (*pal-1*), lethality (*pal-1, tag-331*), larval arrest (*eya-1, tag-331*), uncoordinated movement (*eya-1*), and small size (*sma-4*) [41-44]. Interestingly, the subnetwork comprising of the hub gene *tag-331 *(human ortholog RNF113A) and its 32 direct interactors appears to be largely isolated. A closer examination revealed that two-thirds of these 22 genes are conserved in nematodes yet lack best reciprocal hit orthologs in fly, mouse, or human genomes. The remaining third includes four genes conserved in nematodes, fly, mouse, and human (*zfp-1, R11F4.1, apl-1 *and *fcd-2*) and whose human homologs are linked to disorders (AF10/MLLT10: leukemia, Glycerol kinase: hyperglycerolemia, APP: Alzheimer's, and FANCD2: Fanconi anemia). It remains to be determined if the human genes interact with RNF113A as well as whether RNF113A is involved in any of these diseases. Among the remaining hub genes, the *eya-1 *mammalian orthologs promote development of tissues and organs [41,45,46] whereas the *sma-4 *ortholog SMAD4/DCP4 acts as a tumor suppressor [47-49].

In addition to analyzing the prominent hubs in the interaction network, we also examined the relationship between connectivity of TFs, sequence conservation, and known function. The results revealed a significantly greater number of interactions among *C. elegans *TFs that are conserved in nematodes, fly, mouse, and human, as compared to those that are not (Kruskal-Wallis rank sum test, *p *= 0.0207, after Bonferroni correction). We also found that TF genes associated with mutant phenotypes in RNAi assays exhibit significantly more interactions when compared to those that lack a detectable phenotype (Kruskal-Wallis rank sum test, *p *= 0.0069). These results are consistent with previous studies showing that highly connected hubs tend to be enriched in essential genes [50,51].

## Discussion

This paper presents the first genome-wide comparative study of TF genes in nematodes and their orthologs in fly (*D. melanogaster*), mouse (*M. musculus*), and human (*H. sapiens*). We took both computational and manual curation approaches to compile sets of TF genes in three *Caenorhabditid *species, leading to the identification of 988 genes in *C. elegans*, 995 in *C. briggsae *and 1093 in *C. remanei*. A comparison of these data sets has revealed 652 3-way best reciprocal orthologs among these species. Furthermore, using currently available genome annotations, we identified 150 TF gene orthologs shared among nematodes, fly, mouse, and human and shown that according to mutant phenotypes or associated disorders, many of these genes are functionally important. It should be noted that many of the TF genes identified in *C. elegans *as well as most of those identified as orthologs, paralogs, and divergent in the other two nematode species are based entirely on computational predictions, and thus await experimental validation. However, the results of our study suggest the most likely group of candidate genes from which further experimental tests of TF activity can be designed. In contrast, the majority of the orthologs identified in the two other phyla are annotated as TF genes themselves, owing to the extensive experimental validation performed in these organisms.

The sequence comparison of orthologs among nematodes has revealed that TF genes conserved among the three nematodes species (652 genes) are evolving more rapidly than non-TF genes, which is in agreement with earlier reports from other species in which TF genes have been shown to be evolving more rapidly than the coding genome average, and that significantly more TF genes have been found to be evolving under positive selection when compared to the rest of the genome [24,25,52,53]. While our observation of a greater number of conserved orthologs among all three nematode species, coupled to an accelerated rate of divergence may seem paradoxical, it may be suggestive of widespread positive selection, and thus divergence, acting on genes that are otherwise functionally important. Given the wide estimates of the divergence time between the three nematode species considered in this study, it is unsurprising that the rate of synonymous substitution (*d*_S_) is saturated, and is therefore not amenable for use in analyses that could test the hypothesis of widespread positive selection among TF genes. Additional data, such as a large-scale polymorphism analysis among multiple *Caenorabditid *nematodes could provide the sensitivity to test for evidence of differential selective pressure affecting specific gene groups.

The analysis of TF families in nematodes has revealed several interesting features, such as the high proportion of C2H2 and C4/NHR class of zinc-finger family members relative to the other TF families in all three species (see Figure 3). It was previously shown that the NHR family has undergone significant lineage-specific expansion in *C. elegans *and *C. briggsae *[53]. Considering, for example, that *Drosophila *and humans carry less than 50 identified NHR genes (21 and 48, respectively) [54], the presence of more than 200 genes in *Caenorhabditid *species is striking. Although it remains to be seen whether all of these have important roles to play, studies in *C. elegans *have shown that roughly 10% of NHRs mediate diverse processes including molting (*nhr-23, nhr-25, nhr-67*), neuronal differentiation (*unc-55, fax-1*), sex determination (*sex-1*), and dauer formation (*daf-12*) [54]. We found that roughly half of all NHRs in each of the *Caenorhabditid *species are conserved as 3-way best reciprocal orthologs and another 10% exhibit 2-way orthologous relationships with at least one of the other nematode species. The remaining NHRs are likely to have arisen from lineage-specific gene duplications, suggesting that this class of TF may have a significant role in many of those differences that make individual nematode species unique. While the expansion of the NHR family in nematodes is certainly unusual, other TF families show interesting lineage-specific features as well. Previous studies as well as results presented here indicate that TF families such as ZF-C2H2, HOX and T-box have also diverged between the *C. elegans *and *C. briggsae *lineages (see Figure 3B and additional file 9) [31].

Our work demonstrates that TF genes are non-randomly distributed in the genomes of both *C. elegans *and *C. briggsae*. We found that members of gene families such as NHR, HOX, and T-box are frequently clustered and present in tandem arrays. A subset of the rapidly evolving NHR family of TF genes in *C. elegans *was previously shown to be located on chromosome V [53,55,56]. We have shown not only that *C. briggsae *exhibits a similar pattern, but also that the majority of the chromosome V NHRs in both species is tandemly arrayed. Our finding that many NHRs appear to be lineage-specific paralogs suggests that gene duplication has played a significant role in the expansion of this gene family in nematodes. The phenomenon of gene clustering has been observed not only in *C. elegans*, but also in other species such as *D. melanogaster *and mouse [32-34,55,57], and in some cases these clusters are composed of genes that are co-expressed [32,34]. While the precise mechanism of the origin of such clusters remains to be determined, these may be caused by small-scale regional translocations and illegitimate recombination events leading to tandem gene duplications [58,59].

Our study has revealed that *C. elegans *TF genes conserved across multiple phyla are more likely to be associated with mutant phenotypes when compared to the remaining TF and non-TF genes. Likewise, the fly, mouse, and human orthologs of *C. elegans *TF genes are enriched in essential genes when compared to *C. elegans *TF genes without detectable orthologs (46%, 50% and 23.3%, respectively). The analysis of the relationship between gene function and interactions revealed that TF genes conserved across phyla exhibit greater number of interactions and mutant phenotypes when compared to those that are divergent. Among the TFs with described interactions, *lin-35 *(human *Rb *ortholog) appears to have an exceptionally large number of interactions. *lin-35 *is known to interact with cell cycle-related and chromatin remodeling factors to regulate tissue growth and morphology [60,61]. We found that among the *lin-35 *interacting genes, 43 (8%) encode TFs, of which 18 have best reciprocal hit orthologs in mouse and human. It is important to keep in mind that conservation in sequence does not indicate the roles of orthologous genes in regulating similar biological processes. Instead, it simply means that genes that are evolutionarily conserved are very likely to play important roles in the development and functioning of the organism. Our results are also consistent with studies in other organisms that have found a significant correlation between connectivity, rate of evolution and gene dispensability (according to lethal or sterile phenotype), even across multiple metazoan phyla. In general, hubs with high degree of connectivity tend to be enriched in essential genes and appear to evolve relatively slower than genes with lower connectivity [27,50,62-64].

## Conclusion

This study describes a genome-wide analysis of TF genes in three *Caenorhabditid *nematode species (*C. elegans*, *C. briggsae *and *C. remanei*) as well as their orthologs in fruit fly (*D. melanogaster*), mouse (*M. musculus*) and human (*H. sapiens*). We observed a significantly higher conservation of orthology for the TF genes among *Caenorhabditid *species, while also noting that the coding sequence of TF genes diverges more rapidly than the coding genome average. Finally, the analyses of sequence conservation, gene interactions, and function revealed that TF set conserved in nematodes, fly, mouse, and human is significantly more enriched in essential genes compared to those that lack orthologs in other phyla. Our findings will serve as a resource in aiding us to understand transcriptional networks and their conservation and divergence among metazoa. The compilation of the TF sets also serves as a stepping-stone in generating various resources such as knock-out mutants, cDNA and promoter clones, and reporter gene expressing lines, with the intent of systematically mapping and studying TF function in nematodes. In parallel with many of ongoing initiatives in *C. elegans *these resources will provide foundation for future studies of the conservation of TF function and interaction across the breadth of biodiversity.

## Methods

### *C. elegans*, *C. briggsae *and *C. remanei *TF gene sets

The *C. elegans *TF-encoding genes were searched using 8 GO terms (Table 1) within WS173 release of Wormbase. The *C. briggsae *and *C. remanei *TFs were identified using the HMMER [22,65] and InParanoid programs [21]. The complete genome sequences of each of the three *Caenorhabditid *species were downloaded from WormBase (*C. elegans *release WS173, *C. briggsae *release WS173 and *C. remanei *release 11/29/2005) [17]. As the *C. remanei *predicted peptide dataset is known to contain redundant copies of genes due to heterozygosity in the sequenced genome, (E. Schwartz, personal communication) we used the CD-HIT program (version 2007-0131) [66] in order to cluster and remove all additional transcripts that had greater than or equal to 98% sequence similarity to other transcripts at the protein level. The original dataset of 25,948 transcripts was truncated down to 24,267 non-redundant transcripts that were used in further analysis [27].

InParanoid was run with default values, using blastall version 2.2.14 with –VT emulation, on all three complete genome predicted peptide datasets in pairwise comparisons. The results were collected and placed into species-specific paralogs, 2- and 3-way best-hit reciprocal ortholog categories using custom PERL scripts. Each category was searched for genes from the *C. elegans *TF set and the number of TFs in each category was identified (Additional files 6 and 7). HMM alignment-based searches were carried out on the *C. briggsae *and *C. remanei *predicted peptides using previously established techniques [22,67]. The HMMER signature files (profiles) of known DNA binding domains were retrieved from Pfam [68]. In most cases, a cut-off score of 0.1 was used. If a HMMER predicted TF gene in non-*elegans *species lacked a homolog in *C. elegans*, it was considered false positive and therefore removed creating the final, conservative datasets that were used in the study.

The *C. elegans *orthologs of *D. melanogaster*, *M. musculus *and *H. sapiens *TFs were retrieved using the data available on the InParanoid database [23,69].

### Identification of the TF gene families

Genes were grouped into different families based on the presence of known DNA-binding domains according to the WormBase [14], Pfam [68], and InterPro [70] databases. Only well defined and unambiguous domains that are known to be involved in transcriptional regulation were considered. Families with fewer than 5 members were placed together in a miscellaneous category. The TF families shown in Figure 3 are as follows. AP2: Activator protein-2 family; AT hook: AT hook DNA binding motif (preference to A/T rich region) family; bHLH: basic helix-loop-helix family; bZIP: basic leucine zipper family; CBFB/NF-YA: CCAAT binding factor family; CSD: Cold shock DNA binding domain family; HMG box: High mobility group box family; HOX: Homeobox family; MADF: Myb DNA binding domain family; SAND: DNA binding domain family named after Sp100, AIRE-1, NucP41/75, DEAF-1; SANT: Myb-like DNA binding domain; SMAD: SMAD (Mothers against decapentaplegic (MAD) homolog) domain family; T-box: T-box family; WH: Winged-helix family; WH-FH: Winged-helix and Forkhead domain family; WH-ETS: Winged-helix and ETS domain family; ZF-C2H2: C2H2-type zinc finger protein family; ZF-C2H2-BED: C2H2 and BED-type zinc finger protein family; ZF-BED: BED-type zinc finger family; ZF-C2H2-RING: C2H2 and RING-type zinc finger protein family; ZF-C4/NHR: C4-type zinc finger/Nuclear hormone receptor family; ZF-CCCH: C-x8-C-x5-C-x3-H class of zinc finger family; ZF-DHHC: DHHC-type zinc finger family; ZF-FLYWCH: FLYWCH-type of zinc finger family; ZF-GATA: GATA class of zinc finger family; ZF-PHD: C4HC3 zinc-finger-like motif family; ZF-others: zinc finger family members not listed above; ZF-DM: DM (dsx and mab-3) zinc finger family; ZF, AT hook: AT hook and zinc finger domain family; ZF, SANT: SANT and zinc finger domain family; Misc: Miscellaneous TF family not listed above.

### Generation of the chromosomal map

The physical locations of *C. elegans *and *C. briggsae *TF and non-TF genes were retrieved from Wormbase (WS173 release) and grouped into non-overlapping windows of 200 kb (similar to the 250 kb used by [33]). A 400 kb window analysis was also performed and the conclusions remain the same (data not shown). Since many genes are alternatively spliced, we eliminated transcript-specific bias by focusing on single open reading frame for each transcription factor. In the case of *C. briggsae*, a total of 1329 genes were not assigned to any of the chromosomes and hence were excluded from the analysis. For simplicity, we only used the average between the start and end positions as a proxy for the gene position. The significance of TF clustering on chromosomes was determined by comparing their frequency with the overall frequency of genes in a given window using a χ^2 ^test [33]. Clusters with *p *value less than 0.05 were considered significant.

### Phylogenetic analysis of the nematode NHR genes

The predicted *C. elegans *NHR gene dataset (283 genes) was used to identify orthologs and paralogs in *C. briggsae *and *C. remanei *using the complete genome INPARANOID datasets (see above). 204 and 152 potential homologs were identified in *C. briggsae *and *C. remanei*, respectively. The peptide dataset was aligned using Dialign 2.2 [71] and then manually inspected. We identified two large conserved blocks within most predicted peptides and removed all sequences that did not align within these blocks. The remaining sequences were then realigned with Dialign 2.2 and truncated only to retain the two conserved domains. As per Robinson-Rechavi et al. [29] we chose to use only ungapped sites and removed first sequences missing significant portions of the conserved domains and finally excluded all gapped sites. In the end, we retained 437 sequences (213 *C. elegans*, 106 *C. briggsae *and 118 *C. remanei*) for phylogenetic analysis.

The phylogeny was constructed using a maximum likelihood based method as implemented in PhyML [72] using the JTT substitution model [73] with the default proportion of invariable sites (0.0) and rate heterogeneity between sites corrected by a gamma law (using the default gamma parameter of 1.0 and eight rate categories). The phylogeny was then bootstrapped by generating 1000 randomized datasets using SEQBOOT and assessing the percentage of consensus trees using CONSENSE, both in the PHYLIP package [74].

### Calculation of TF divergence

DNA sequences from *C. elegans*, *C. briggsae *and *C. remanei *were aligned according to their protein alignment using Dialign 2.2 [75] and RevTrans 1.4 [76]. Rates of synonymous substitutions per synonymous site (*d*_*S*_) and non-synonymous substitutions per non-synonymous site (*d*_*N*_) were estimated using codeml from PAML [77]. Evolutionary rates between TF and non-TF data sets were compared using a permuted Kruskal-Wallis rank sum test using 10,000 permutations.

### Curation of the mutant phenotypes of TFs

The RNAi phenotypes of all known *C. elegans *genes were retrieved from Wormbase (WS170 release). A total of 13,648 phenotypes associated with 4,351 genes were analyzed and sorted into 82 different categories (Unc, Dpy, Vul etc.) (Additional files 13 and 14).

For phenotypes associated with *C. elegans *TF orthologs in fly, mouse, and human, we searched Flybase [78], NCBI OMIM [79], PubMed [80], and other public databases (, , , ). Only those phenotypes that were unambiguous and did not show discrepancy between different published sources were included. In order to reduced any effect linked to a differential amount of genes annotated as involved in particular mutant phenotypes, all the analyses were performed within each phenotypic class by comparing the distribution of genes with mutant phenotypes among the different sets (non-TF genes, TF genes, *C. elegans *TF genes, TF genes conserved among the three nematode species, and TF genes with orthologs in nematodes, fly, mouse, and human).

### Construction of TF interaction network

The *C. elegans *gene network was built using the genetic and protein-protein interaction data for transcription factors curated by BioGRID (version 2.0.27 release) [37,38]. The network was visualized by using Cytoscape [81].

## Abbreviations

*d*_N:_ non-synonymous substitutions per non-synonymous site; *d*_S:_ synonymous substitutions per synonymous site; NHR: Nuclear hormone receptor; TF: Transcription factor; ZF: Zinc finger.

## Authors' contributions

The laboratories of BPG and RSS contributed to this publication. BPG and NK identified the *C. elegans *TF set, protein families, chromosomal maps, and mutant phenotypes. WH identified nematode TF orthologs in fly, mouse, and human and carried out most of the sequence alignments, and interaction network analysis. CA performed the InParanoid orthology searches creating the *C. briggsae *and *C. remanei *TF gene sets and constructed NHR phylogenetic tree. BPG, WH and CA drafted the manuscript. BPG conceived and coordinated the study. All authors read and approved the final manuscript.

## Supplementary Material

Additional file 1 List of 314 incorrect entries (non-TFs) in *C. elegans*. Click here for file

Additional file 2 List of 167 genes that encode chromatin remodeling, general transcription and DNA/RNA binding factors in *C. elegans*. Click here for file

Additional file 3 List of 83 histone-encoding genes in *C. elegans*. Click here for file

Additional file 4 List of 988 TF genes in *C. elegans*. Click here for file

Additional file 5 List of 17 false positives in wTF2.0. Click here for file

Additional file 6 List of 995 TF genes in *C. briggsae*. Click here for file

Additional file 7 List of 1093 TF genes in *C. remanei*. Click here for file

Additional file 8 List of 150 TF orthologs in *Drosophila melanogaster*, *M. musculus*, and *H. sapiens*. Click here for file

Additional file 9 BLASTP hits of *C. briggsae*-divergent TFs in *C. elegans *genome. Click here for file

Additional file 10 Tandem arrays of TF genes in *C. elegans*. Click here for file

Additional file 11 Tandem arrays of TF genes in *C. briggsae*. Click here for file

Additional file 12 Phylogenetic tree of NHR genes in *Caenorhabditid *nematode species. Colors mark NHR genes in different species (blue: *C. elegans*, red: *C. briggsae *and light green: *C. remanei*). Tandemly along chromosomes and phylogenetically clustered genes are indicated by vertical bars. Chromosomes carrying NHR clusters are indicated by roman numerals. The sub-branch comprised of seven groups of NHR genes on chromosome V has been marked by a star (*). Scale bar represents 0.5 substitutions per site. Click here for file

Additional file 13 *C. elegans *genes and their mutant phenotypes in RNAi assays. Click here for file

Additional file 14 RNAi phenotypes sorted into six broad categories. Click here for file

Additional file 15 List of the worm (*C. elegans*) fly (*D. melanogaster*) and mouse (*M. musculus*) mutant phenotypes associated with conserved TF genes. Click here for file

Additional file 16 List of TF genes and their interactors. The columns A and B merely list gene pairs that interact with each other and do not indicate the direction of regulation. Click here for file
