# Supplementary figures and images for: Comparative analysis of function and interaction of transcription factors in nematodes: Extensive conservation of orthology coupled to rapid sequence evolution
Source: BMC Genomics. 2008 Aug 27;9:399. doi: 10.1186/1471-2164-9-399 (PMC2533025; doi:10.1186/1471-2164-9-399)

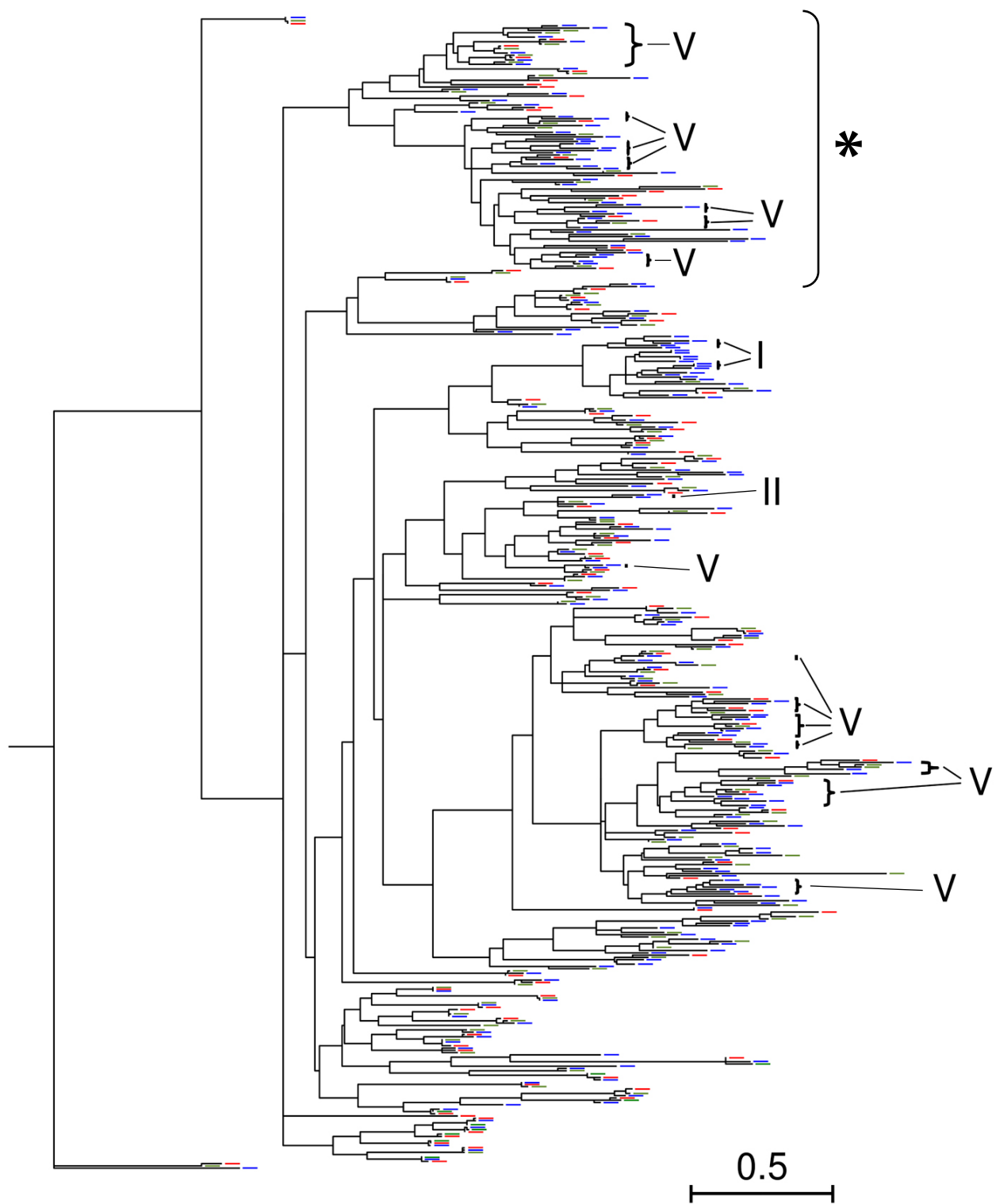

Supplement: Additional file 12 — Phylogenetic tree of NHR genes in Caenorhabditid nematode species. Colors mark NHR genes in different species (blue: C. elegans, red: C. briggsae and light green: C. remanei). Tandemly along chromosomes and phylogenetically clustered genes are indicated by vertical bars. Chromosomes carrying NHR clusters are indicated by roman numerals. The sub-branch comprised of seven groups of NHR genes on chromosome V has been marked by a star (*). Scale bar represents 0.5 substitutions per site. [file 1471-2164-9-399-S12.pdf]
