# Supplementary material for: Comparative analysis of function and interaction of transcription factors in nematodes: Extensive conservation of orthology coupled to rapid sequence evolution
Source: BMC Genomics. 2008 Aug 27;9:399. doi: 10.1186/1471-2164-9-399 (PMC2533025; doi:10.1186/1471-2164-9-399)
Supplement: Additional file 14 — RNAi phenotypes sorted into six broad categories. [file 1471-2164-9-399-S14.pdf]

Additional file 14.

Classification of RNAi phenotypes into six broad biological categories.

			Biological category					
**RNAi class**	**RNAi phenotype**	**WB RNAi class**	**Fertility**	**Viability**	**Vulva**	**Sex**	**Body**	**Behavior**
Abs	spindle_position_abnormal_early_emb	[WBPhenotype0000762]		x				
Adl	adult_early_lethal	[WBPhenotype0000060]		x				
Age	life_span_abnormal	[WBPhenotype0000039]						
Bli	blistered	[WBPhenotype0000025]					x	
Bmd	organism_morphology_abnormal	[WBPhenotype0000535]					x	
Cda	cell_division_abnormal	[WBPhenotype0000746]						
Ced	cell_death_abnormal	[WBPhenotype0000729]						
Clr	clear	[WBPhenotype0001010]					x	
Con	constipated	[WBPhenotype0000651]						x
Cyk	cytokinesis_abnormal	[WBPhenotype0001018]						
Daf	dauer_formation_abnormal	[WBPhenotype0000637]						
Dpy	dumpy	[WBPhenotype0000583]					x	
Eat	feeding_behavior_abnormal	[WBPhenotype0000659]						x
Egl	egg_laying_abnormal	[WBPhenotype0000640]				x		
Egl_D	egg_laying_defective	[WBPhenotype0000006]				x		
Ela	early_larval_arrest	[WBPhenotype0000055]		x				
Emb	egg_size_abnormal_early_emb	[WBPhenotype0000044]		x				
Emo	endomitotic_oocytes	[WBPhenotype0000668]						
Esp	pathogen_susceptibility_increased	[WBPhenotype0001013]						
Evl	everted_vulva	[WBPhenotype0000696]			x	x		
Fem	feminization_of_XX_and_XO_animals	[WBPhenotype0000687]				x		
Fgc	fewer_germ_cells	[WBPhenotype0000684]	x					
Fog	feminization_of_germline	[WBPhenotype0000682]				x		
Gab	gastrulation_abnormal	[WBPhenotype0000047]						
gda	germ_cell_development_abnormal	[WBPhenotype0000812]	x			x		

gdev	gonad_development_abnormal	[WBPhenotype0000691]				x		
glp	germ_cell_proliferation_abnormal	[WBPhenotype0000823]	x					
Gom	gonad_migration_abnormal	[WBPhenotype0000690]				x		
Gro	slow_growth	[WBPhenotype0000031]		x				
Him	high_incidence_male_progeny	[WBPhenotype0001175]						
Hya	hyperactive	[WBPhenotype0000642]						x
Let	larval_lethal	[WBPhenotype0000054]		x				
Let-L1	L1_arrest	[WBPhenotype0000081]		x				
Let-L2	L2_arrest	[WBPhenotype0000082]		x				
Let-L3	L3_arrest	[WBPhenotype0000083]		x				
Lin	lineage_abnormal	[WBPhenotype0000093]						
Lla	late_larval_arrest	[WBPhenotype0000056]		x				
Lon	long	[WBPhenotype0000022]					x	
Lva	larval_arrest	[WBPhenotype0000059]		x				
Mab	male_morphology_abnormal	[WBPhenotype0001024]					x	
Mec	mechanosensory_abnormal	[WBPhenotype0000315]						x
Mei	meiosis_abnormal_early_emb	[WBPhenotype0001041]		x				
Mel	maternal_effect_lethal_emb	[WBPhenotype0000052]		x				
Mig	cell_migration_abnormal	[WBPhenotype0000594]						
Mlt	molt_defect	[WBPhenotype0000638]						
Mog	masculinization_of_germline	[WBPhenotype0000683]				x		
Mul	multiple_nuclei_early_emb	[WBPhenotype0001143]		x				
Muv	multivulva	[WBPhenotype0000700]			x	x		
Nmo	nuclear_morphology_alteration_early_emb	[WBPhenotype0001026]		x				
Npo	nuclear_position_abnormal_early_emb	[WBPhenotype0001027]		x				
Ocs	one_cell_arrest_early_emb	[WBPhenotype0000040]		x				
Oma	oocyte_meiotic_maturation_abnormal	[WBPhenotype0000105]	x					
Pat	paralyzed_arrested_elongation_at_two_fold	[WBPhenotype0000053]						x
pBoc	pos_body_wall_contraction_abnormal	[WBPhenotype0000157]						x
Pch	patchy_coloration	[WBPhenotype0001029]					x	
P-granule	p_granule_localization_abnormal	[WBPhenotype0001302]	x					
Pna	pronuclear_envelope_abnormal_early_emb	[WBPhenotype0001030]		x				

Prl	paralyzed	[WBPhenotype0000644]						x
Pro	proximal_germ_cell_proliferation_abnormal	[WBPhenotype0001033]	x					
Pvl	protruding_vulva	[WBPhenotype0000697]			x	x		
Rbs	reduced_brood_size	[WBPhenotype0000154]	x					
Rde	RNAi_response_abnormal	[WBPhenotype0000743]						
Ric	aldicarb_resistant	[WBPhenotype0000017]						x
Rol	roller	[WBPhenotype0000645]						x
Rot	centrosome_pair_&_assoc_pronuclear_rotation_ab	[WBPhenotype0000766]						
Rup	exploded_through_vulva	[WBPhenotype0000038]			x	x		
Sck	sick	[WBPhenotype0000032]						x
Sle	slow_embryonic_development	[WBPhenotype0000042]		x				
SlowGro	developmental_delay_postembryonic	[WBPhenotype0000045]		x				
Slu	sluggish	[WBPhenotype0000646]						x
Sma	small	[WBPhenotype0000229]					x	
Spd	spindle_abnormal_early_emb	[WBPhenotype0000759]		x				
Spn	spindle_orientation_abnormal_early_emb	[WBPhenotype0000760]		x				
Spo	spindle_position_orientation_abnormal_early_emb	[WBPhenotype0000761]		x				
Sqt	squat	[WBPhenotype0000021]					x	
Ste	sterile	[WBPhenotype0000688]	x					
Stp	sterile_progeny	[WBPhenotype0001037]	x					
Thin	thin	[WBPhenotype0000164]					x	
Tum	tumorous_germline	[WBPhenotype0001038]	x					
unc	locomotion_abnormal	[WBPhenotype0000643]						x
Vul	vulvaless	[WBPhenotype0000698]			x	x		
VulAb	vulva_cell_fate_specification_abnormal	[WBPhenotype0000220]			x	x		

Fertility: Germ cell and germline-related defects, Viability: Embryonic and post-embryonic growth and survival-related defects, Vulva: Vulval cell proliferation and morphogenesis defects, Sex: Sex determination and reproductive system-related defects, Body: Body size, cuticle, and morphology-related defects, Behavior: Movement and feeding-related defects.
